# A New Phenotypic Expression in a Patient With a Mutation in the CACNA1F Gene

**DOI:** 10.7759/cureus.82577

**Published:** 2025-04-19

**Authors:** Ricardo A Murati Calderon, Natalio Izquierdo

**Affiliations:** 1 Department of Ophthalmology, School of Medicine, University of Puerto Rico, Medical Sciences Campus, San Juan, PRI; 2 Department of Surgery, School of Medicine, University of Puerto Rico, Medical Sciences Campus, San Juan, PRI

**Keywords:** calcium channel, cone-rod dystrophy, inherited retinal dystrophies, night blindness, retinitis pigmentosa (rp), rod-cone dystrophy, x-linked genetic diseases

## Abstract

Mutations in the *CACNA1F* gene are associated with various X-linked retinal disorders, including congenital stationary night blindness type 2A (CSNB2A), cone-rod dystrophy (CORDX3), and Åland Island eye disease (AIED), due to their role in calcium channel function in retinal photoreceptor synapses. In this report, we present the case of a 33-year-old Hispanic male patient with childhood-onset nyctalopia and progressive visual loss. Fundus examination revealed optic disc pallor and cupping, vascular attenuation, chorioretinal atrophy, and mid-peripheral bony spicules. Full-field electroretinography (ERG) demonstrated severely reduced scotopic and photopic responses, with non-discernible a- and b-waves and significantly diminished 30 Hz flicker amplitudes with delayed peak times. Humphrey visual field testing showed bilateral peripheral field constriction. A clinical diagnosis of retinitis pigmentosa was made. Genetic testing via next-generation sequencing revealed a hemizygous pathogenic mutation in *CACNA1F*, specifically c.5037_5038del (p.Leu1681Alafs*16), leading to a truncated, non-functional protein. While this variant has been reported in genetic databases, detailed phenotypic descriptions remain scarce. Our findings are most consistent with cone-rod dystrophy, although visual field defects also overlap with features of AIED. This case highlights the phenotypic heterogeneity of *CACNA1F*-related disorders and suggests rod-cone dystrophy as a potential additional phenotype. Further studies are warranted to clarify the full clinical spectrum and molecular mechanisms associated with *CACNA1F* mutations.

## Introduction

In 1998, Strom and colleagues first described the *CACNA1F* gene located on the X chromosome [1]. This gene encodes the α1F subunit of the voltage-dependent calcium channel, CaV1.4, essential for calcium ion influx in retinal photoreceptors [2]. This channel is critical for the synaptic transmission of visual signals from photoreceptors to bipolar cells, ensuring adequate retinal function [3]. Mutations in the *CACNA1F* gene disrupt this pathway, leading to a spectrum of X-linked retinal disorders that exhibit overlapping but distinct phenotypic features [4].

Three significant phenotypes associated with mutations in the *CACNA1F* gene have been well-described: congenital stationary night blindness type 2A (CSNB2A) [5,6], cone-rod dystrophy (CORDX3) [7], and Åland Island eye disease (AIED) [4]. Each presentation has distinct electroretinographic and visual field findings, which help in the differential diagnosis.

Patients with CSNB2A have impaired night vision, congenital nystagmus, and variable visual acuity, often without significant structural retinal abnormalities [8]. While no definitive population-level prevalence has been established, a large meta-analysis of over 470 confirmed cases indicated that X-linked forms such as CSNB2A account for approximately 57.9% of cases, followed by autosomal recessive (40%) and autosomal dominant (2.1%) [9]. Patients usually have minimal fundoscopic changes, making functional testing such as electroretinography (ERG) essential for diagnosis. In ERG, patients may have a negative ERG pattern, showing a normal scotopic a-wave with an attenuated or absent b-wave [5]. The b-wave findings reflect a transmission defect at the photoreceptor-to-bipolar cell synapse [10]. Visual field findings are generally unremarkable in most patients [5].

In contrast, patients with CORDX3 have progressive degeneration of both cone and rod photoreceptors, resulting in structural and functional retinal dysfunction over time. Cone-rod dystrophies are rare, with an estimated incidence of 1 in 20,000 to 100,000 [11]. Clinical features include diminished visual acuity, photophobia, impaired color vision, and central scotomas [7]. ERG findings in these patients include markedly reduced or non-recordable photopic responses, indicating cone dysfunction; reduced scotopic a- and b-waves, reflecting rod involvement; and abnormal 30 Hz flicker responses with reduced amplitudes and prolonged peak times, reflecting a defective synaptic transmission [12]. Visual field defects, such as central scotomas or paracentral defects, may be observed and are associated with cone degeneration [13]. As the disease progresses, peripheral field constriction may also occur with rod photoreceptor dysfunction [14].

Patients with AIED have high myopia, astigmatism, nystagmus, reduced visual acuity, retinal hypopigmentation, and abnormal ERG findings [15]. This entity manifests its features early in life and has an estimated birth prevalence of approximately 1 in 22,000 live-born males, according to a cohort study that included a Danish population [4,16]. It shares overlapping features with CSNB2A, particularly defective dark adaptation, but can be distinguished by structural abnormalities in the macula, particularly foveal hypoplasia [16]. ERG findings in patients with AIED typically show significantly reduced b-waves, consistent with a classic negative ERG. However, the main defining feature is the presence of foveal hypoplasia.

Maddox and coworkers, in 2020, described that the *CACNA1F* gene product encodes a multi-pass transmembrane protein that forms a complex with multiple subunits in a 1:1:1:1 ratio, regulating calcium signaling at the photoreceptor synapse [3]. This calcium channel plays a role in sustaining neurotransmitter release from photoreceptors to bipolar cells, ensuring continuous signal transmission under varying light conditions [17].

Given its critical function in synaptic transmission, even subtle alterations in the channel can disrupt retinal signaling and lead to a range of clinical manifestations. Alternatively, spliced transcript variants encoding multiple isoforms have been identified, leading to structural and functional variations in the channel [18]. These isoforms may influence the voltage dependence, inactivation properties, and synaptic transmission efficiency of the channel, ultimately contributing to the phenotypic diversity observed in *CACNA1F*-related disorders [18]. Consequently, patients with mutations in *CACNA1F* may exhibit a broad spectrum of retinal pathologies, ranging from CSNB2A to progressive degenerative diseases such as CORDX3. The overlapping clinical features, variable severity, and phenotypic continuum of these disorders pose a diagnostic challenge, often requiring advanced electrophysiological testing and genetic analysis to differentiate them [15].

Here, we report on a 33-year-old male patient with a new phenotypic manifestation caused by a hemizygous pathogenic mutation in the *CACNA1F* gene.

## Case presentation

A 33-year-old Hispanic male patient with a past medical history of hypothyroidism and myopia was referred to our local clinic for genetic evaluation. The patient had nyctalopia since childhood. There was no family history of eye disease. Upon comprehensive ophthalmic evaluation by at least one of the authors, his best corrected visual acuity (BCVA) was 20/50 in both eyes (OU). Refraction was -13.25 +1.50 x 100˚ in the right eye (OD) and -12.50 +1.00 x 96˚ in the left eye (OS). Intraocular pressure with applanation tonometry was 14 mmHg in the right eye and 13 mmHg in the left eye. The pupils were round and reactive to light, and there was no afferent pupillary defect in both eyes. Upon Hardy-Rand-Rittler (HRR) pseudoisochromatic testing, the patient had a mild red-green color deficiency. The external examination was remarkable for alternating horizontal nystagmus. Slit lamp examination was unremarkable in both eyes. Upon fundus examination, the patient had bilateral optic disc pallor and cupping, arteriolar attenuation, and mid-peripheral bony spicules.

**Figure 1:**
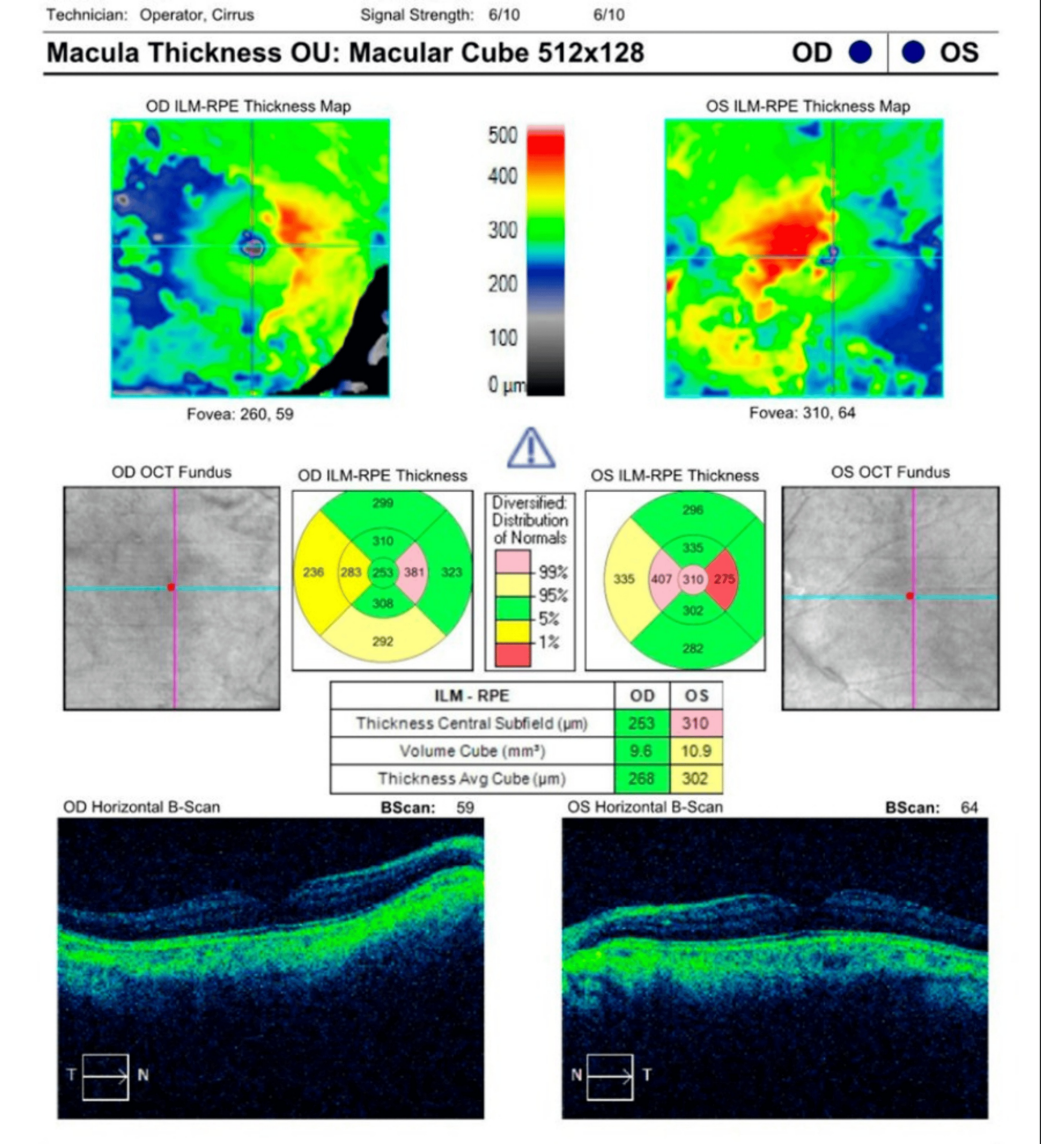
Macular stratus optical coherence tomography shows decreased macular thickness and volume in the right eye (OD). OCT: optical coherence tomography, RPE: retinal pigment epithelium, ILM: inner limiting membrane

**Figure 2:**
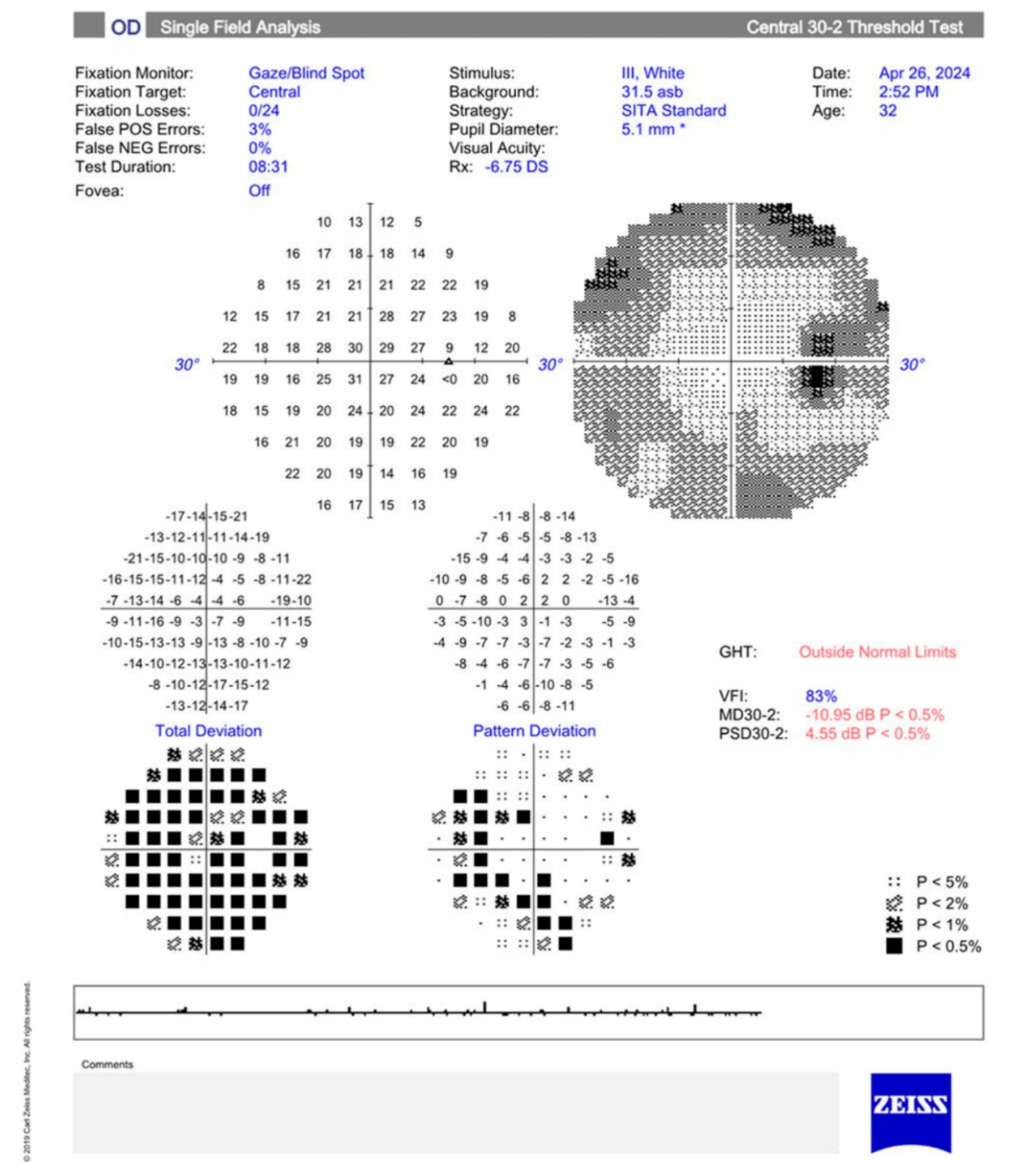
Visual field testing (30-2; Carl Zeiss Meditec AG) shows a significantly decreased mean deviation (p<0.5%) in the right eye. GHT: glaucoma hemifield test, VFI: visual field index, MD: mean deviation, PSD: pattern standard deviation

**Figure 3:**
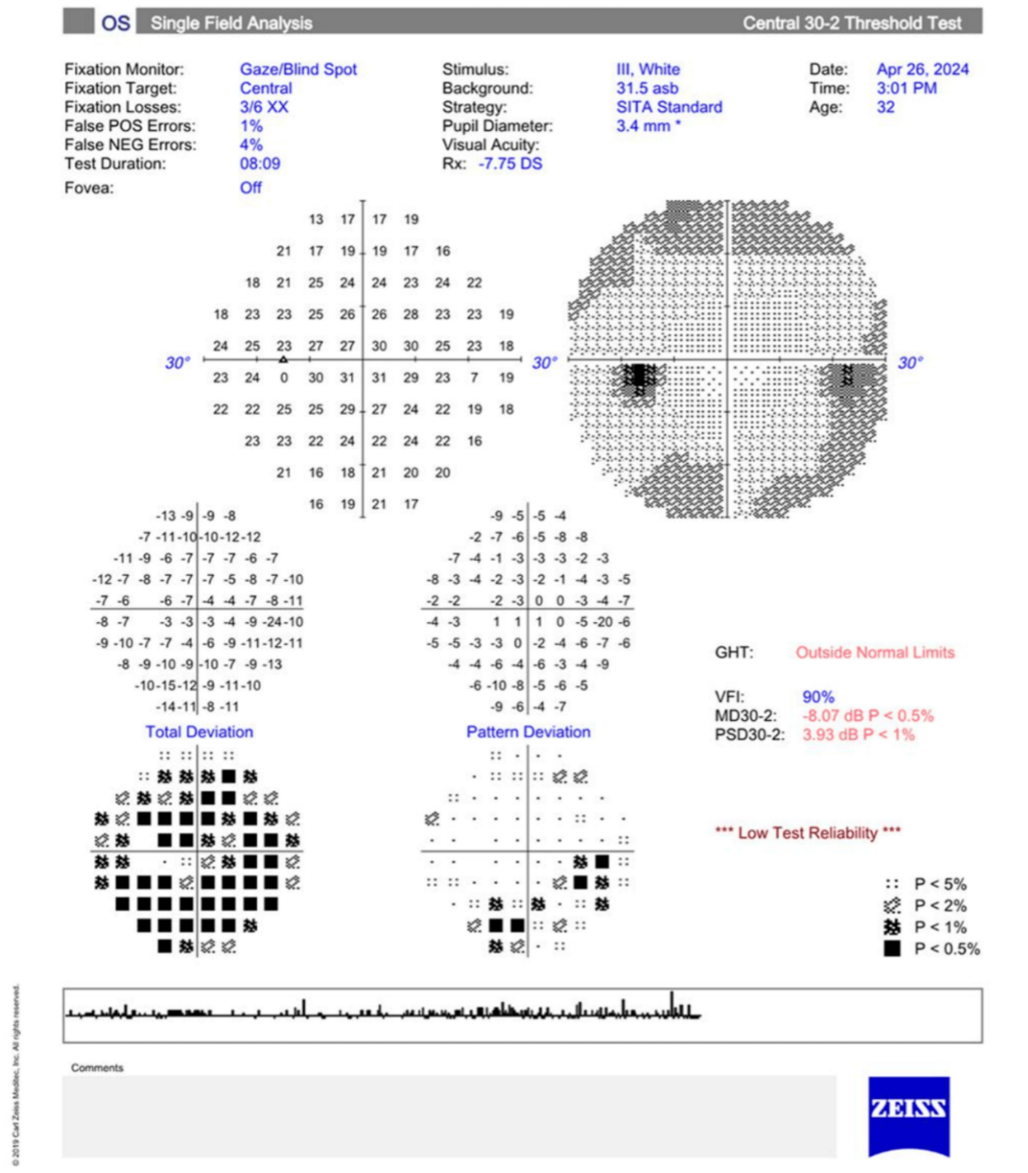
Visual field testing (30-2; Carl Zeiss Meditec AG) shows a significantly decreased mean deviation (p<0.5%) in the left eye. GHT: glaucoma hemifield test, VFI: visual field index, MD: mean deviation, PSD: pattern standard deviation

As depicted in Figure 1, macular stratus optic coherence tomography (OCT) showed that the patient had a macular volume of 9.6 mm^3^ and 10.9 mm^3^ in the right and left eye, respectively. The average macular thickness was 268 µm and 302 µm in the right and left eye, respectively. There was no inner segment/outer segment border loss. Visual field examination using the Humphrey test (30-2; Carl Zeiss Meditec AG, Germany) showed a mean deviation of -10.95 dB (p < 0.5%) in the right eye and -8.07 dB (p < 0.5%) in the left eye. The pattern standard deviation was 4.55 dB (p < 0.5%) in the right eye and 3.93 dB (p < 0.5%) in the left eye, as shown in Figures 2-3.

Upon full-field electroretinography, our patient showed nearly extinguished scotopic and photopic responses bilaterally, with undetectable a- and b-waveforms. Responses to the 30 Hz flicker showed reduced amplitudes and abnormally slow peak times.

A clinical diagnosis of retinitis pigmentosa (RP) was reached. Genetic testing was performed using a saliva sample, and a next-generation sequencing (NGS) diagnostic test (Laboratory for Molecular Medicine, Center for Genetics and Genomics, Cambridge, USA) was conducted to evaluate over 300 genes associated with inherited retinal genetic diseases. The results showed a hemizygous pathogenic mutation in the *CACNA1F* gene, specifically with a variant of c.5037_5038del (p.Leu1681Alafs*16). As part of the management plan, the patient was initiated on lutein and zeaxanthin supplementation and counseled on avoiding toxic habits such as consuming alcohol and tobacco. Upon follow-up, the patient has remained clinically stable.

## Discussion

Previous studies have reported that patients with *CACNA1F* mutations may have phenotypic presentations, including CSNB2A, CORDX3, and AIED [4,5,7]. Patients with CSNB2A have a characteristic negative ERG with a preserved a-wave and attenuated b-wave [5,9]. Our patient’s ERG showed the typical a- and b-waveforms that were not discernible from the scotopic and photopic ERG responses. Responses to the 30 Hz flicker had reduced amplitudes and abnormally slow peak times. The oscillatory potential was diminished. These findings differ from CSNB2A.

Studies by Jalkanen et al. and Hauke et al. have characterized the classic electroretinographic patterns associated with CORDX3 in patients with mutations in *CACNA1F* [7,12]. These patients have markedly reduced or non-recordable photopic responses, reduced scotopic a- and b-waves, and abnormal 30 Hz flicker responses with reduced amplitudes and prolonged peak times, reflecting a defective synaptic transmission [7,12]. Our patient’s ERG showed non-discernible a- and b-waveforms in the scotopic and photopic waves. Responses to the 30 Hz flicker had reduced amplitudes and abnormally slow peak times. Findings in our patient are incompatible with CACNA1F-associated CORDX3 due to non-discernible a- and b-waveforms in the scotopic wave.

Our patient had high myopia, which occurs in patients with AIED. However, Wyględowska-Promieńska et al., in 2024, showed that ERG results in patients with AIED resemble the negative ERG seen in CSNB2A [15]. Our patient had absent photopic and scotopic responses. Hence, our patient’s ERG findings are incompatible with AIED.

Bijveld et al., in 2013, described patients with CSNB2A as typically having adequate peripheral visual fields [5]. Our patient had peripheral visual field defects. These findings help confirm that our patient did not have CSNB2A.

Gill et al., in 2019, discussed visual field findings in patients with progressive cone and cone-rod dystrophies [11]. Central scotomas and generalized reduction in sensitivity in the central field were typical of cone dysfunction, which may be followed by progressive involvement of rod photoreceptors, ultimately leading to peripheral vision loss [11]. Our patient had peripheral visual field defects and unremarkable central field findings upon visual field testing. These visual field results are compatible with rod-cone dystrophy. To our knowledge, this case represents the first report of visual field findings compatible with rod-cone dystrophy associated with mutations in *CACNA1F*. In contrast, patients with AIED often exhibit peripheral field constriction due to progressive rod dysfunction [15,19]. Our patient had peripheral field defects. Even though visual field findings are compatible with AIED, ERG findings help differentiate between these clinical presentations.

To facilitate phenotype differentiation for future diagnostic guidance, we have summarized the clinical and diagnostic features of CSNB2A, CORDX3, and AIED alongside our patient’s findings (Table 1).

**Table 1:** Comparison of clinical and diagnostic features among CACNA1F-related phenotypes CSNB2A: congenital stationary night blindness type 2A; CORDX3: cone-rod dystrophy X-linked 3; AIED: Åland Island eye disease; ERG: electroretinography; OCT: optical coherence tomography; RPE: retinal pigment epithelium; VF: visual field

Feature	CSNB2A	CORDX3	AIED	Our patient
Night vision	Impaired since birth	Progressive nyctalopia	Impaired	Impaired since childhood
Visual acuity	Variable, often stable	Progressive decline	Decreased early in life	20/50 OU
Nystagmus	Common	Occasionally present	Common	Alternating horizontal
Fundus findings	Often normal	Disc pallor, RPE changes	Fundus hypopigmentation	Bony spicules, disc pallor
ERG	Negative (normal a-, ↓b-wave)	↓↓ photopic & scotopic, ↓ flicker	Negative (normal a-,↓b-wave)	Non-discernible a-/b-waves, ↓ flicker
OCT	Typically normal	Outer retinal thinning	Foveal hypoplasia	Decreased macular thickness (OD)
Visual field	Normal	Central ± peripheral defects	Peripheral constriction	Peripheral VF defects
Color vision	Often mildly reduced	Red-green defects	Protan anomalies	Red-green deficiency

Beyond the three classic phenotypic presentations described, several studies further illustrate the heterogeneity of CACNA1F-related retinal disorders. Mihalich et al. described two novel mutations producing clinically distinct phenotypes, one consistent with CSNB2A and the other with AIED [4]. This report highlights the variability in ERG and structural findings despite mutations in the same gene [4]. Similarly, Wyględowska-Promieńska et al. described a negative ERG pattern in a case of AIED associated with retinoschisis [15]. Lastly, Vincent and colleagues, in 2011, described a novel missense mutation in the *CACNA1F *gene, identified in individuals from the same family who presented with distinct phenotypes consistent with AIED and CSNB [19]. These reports emphasize the broad phenotypic spectrum of CACNA1F mutations and support the relevance of detailed case characterization.

Several mutations in the *CACNA1F* gene have been reported. Our patient's genetic testing confirmed a hemizygous pathogenic frameshift mutation, c.5037_5038del (p.Leu1681Alafs*16), in the *CACNA1F* gene. Frameshift deletions are particularly deleterious as they result in premature truncation of the protein, disrupting the function of the CaV1.4 calcium channel and abolishing synaptic transmission between photoreceptors and bipolar cells [18]. This specific mutation has been previously documented among variants associated with inherited retinal diseases in an extensive genetic cohort study. However, detailed phenotypic descriptions of patients with this mutation have not been reported. Our case contributes valuable clinical correlation to this variant, further defining its role within the phenotypic spectrum of *CACNA1F*-related retinal dystrophies.

Clinical findings in our patient show phenotypic heterogeneity in patients with mutations in *CACNA1F*. Furthermore, we propose that patients with mutations in this gene may have rod-cone dystrophy as a fourth clinical presentation.

This case highlights the importance of genetic testing in patients with atypical retinal findings. Phenotypic presentation alone may not be sufficient to classify *CACNA1F*-related disorders. A multimodal diagnostic approach is recommended when evaluating patients with inherited retinal diseases, especially those with *CACNA1F* mutations.

This report's limitations include the lack of longitudinal follow-up and the absence of detailed family segregation analysis to determine inheritance patterns. Future studies are needed to determine whether rod-cone dystrophy represents a distinct fourth phenotype within the CACNA1F spectrum or reflects the overlapping phenotypic evolution from existing classifications.

## Conclusions

This case contributes to the phenotypic spectrum associated with *CACNA1F *mutations by presenting the case of a 33-year-old male patient with clinical and functional findings consistent with rod-cone dystrophy, a phenotype not classically associated with this gene. Unlike previously described presentations of CSNB2A, CORDX3, and AIED, our patient demonstrated a combination of peripheral visual field constriction, extinguished ERG responses, and progressive nyctalopia, suggestive of rod-predominant dysfunction. Identifying the pathogenic c.5037_5038del (p.Leu1681Alafs*16) frameshift variant further supports a severe functional impact on CaV1.4 channel activity. To our knowledge, this is the first report correlating this specific mutation with a rod-cone dystrophy presentation.

These findings demonstrate the clinical variability of *CACNA1F*-related retinal disease and highlight the essential role of comprehensive genetic testing and electrophysiological studies in achieving diagnostic accuracy. Importantly, this case may guide clinicians in recognizing underdiagnosed presentations and contribute to more precise genotype-phenotype correlations for *CACNA1F*.
